# 5,13-Disulfamoyl-1,9-diazatetracyclo[7.7.1.0^2,7^.0^10,15^]heptadeca-2(7),3,5,10,12,14-hexaen-1-ium chloride

**DOI:** 10.1107/S1600536811038189

**Published:** 2011-09-30

**Authors:** Yichao Xu, Shouwen Jin, Jianlong Zhu, YingJia Liu, ChuanChuan Shi

**Affiliations:** a Tianmu College of ZheJiang A & F University, Lin’An 311300, People’s Republic of China

## Abstract

In the title salt, C_15_H_17_N_4_O_4_S_2_^+^·Cl^−^, the chloride anion is disordered over two positions with occupancies of 0.776 (6) and 0.224 (6). The cation adopts an L shape and the dihedral angle between the benzene rings is 82.5 (3)°. In the crystal, inversion dimers of cations linked by pairs of N—H⋯N hydrogen bonds occur, with the bond arising from the protonated N atom. The cationic dimers are linked into chains *via* the disordered chloride ions by way of N—H⋯Cl hydrogen bonds and N—H⋯O, C—H⋯O and C—H⋯Cl inter­actions also occur, which help to consolidate the three-dimensional network.

## Related literature

For a related structure and background references to supra­molecular networks, see: Jin *et al.* (2010 ▶).
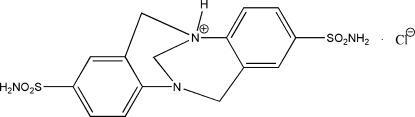


## Experimental

### 

#### Crystal data

C_15_H_17_N_4_O_4_S_2_^+^·Cl^−^
*M* *_r_* = 416.90
Monoclinic, 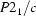

*a* = 11.5247 (11) Å
*b* = 18.5693 (16) Å
*c* = 8.1489 (7) Å
β = 109.177 (1)°
*V* = 1647.1 (3) Å^3^
*Z* = 4
Mo *K*α radiation
μ = 0.52 mm^−1^
*T* = 298 K
0.40 × 0.33 × 0.27 mm

#### Data collection

Bruker SMART CCD diffractometer
Absorption correction: multi-scan (*SADABS*; Bruker, 2002 ▶) *T*_min_ = 0.815, *T*_max_ = 0.869
8082 measured reflections
2895 independent reflections
1944 reflections with *I* > 2σ(*I*)
*R*_int_ = 0.042

#### Refinement

*R*[*F* ^2^ > 2σ(*F* ^2^)] = 0.068
*wR*(*F* ^2^) = 0.219
*S* = 1.08
2895 reflections
239 parameters
H-atom parameters constrained
Δρ_max_ = 0.52 e Å^−3^
Δρ_min_ = −0.96 e Å^−3^

### 

Data collection: *SMART* (Bruker, 2002 ▶); cell refinement: *SAINT* (Bruker, 2002 ▶); data reduction: *SAINT*; program(s) used to solve structure: *SHELXS97* (Sheldrick, 2008 ▶); program(s) used to refine structure: *SHELXL97* (Sheldrick, 2008 ▶); molecular graphics: *SHELXTL* (Sheldrick, 2008 ▶); software used to prepare material for publication: *SHELXL97*.

## Supplementary Material

Crystal structure: contains datablock(s) global, I. DOI: 10.1107/S1600536811038189/hb6395sup1.cif

Structure factors: contains datablock(s) I. DOI: 10.1107/S1600536811038189/hb6395Isup2.hkl

Supplementary material file. DOI: 10.1107/S1600536811038189/hb6395Isup3.cml

Additional supplementary materials:  crystallographic information; 3D view; checkCIF report

## Figures and Tables

**Table 1:** Hydrogen-bond geometry (Å, °)

*D*—H⋯*A*	*D*—H	H⋯*A*	*D*⋯*A*	*D*—H⋯*A*
N1—H1*A*⋯O2^i^	0.89	2.26	3.081 (8)	153
N1—H1*B*⋯Cl1^ii^	0.89	2.26	3.141 (7)	170
N2—H2*A*⋯Cl1^iii^	0.89	2.19	3.042 (6)	160
N2—H2*B*⋯O1^iv^	0.89	2.46	3.109 (7)	130
N2—H2*B*⋯O2^iv^	0.89	2.36	3.240 (7)	168
N4—H4⋯N2^v^	0.91	2.02	2.922 (7)	173
C12—H12⋯O3^vi^	0.93	2.49	3.416 (7)	175
C13—H13*A*⋯Cl1^vii^	0.97	2.76	3.514 (7)	135
C14—H14*A*⋯O4^i^	0.97	2.50	3.212 (9)	130
C15—H15*A*⋯O3^viii^	0.97	2.52	3.443 (7)	158

Symmetry codes: (i) 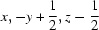; (ii) 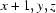; (iii) 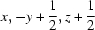; (iv) 
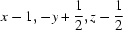
; (v) 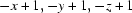; (vi) 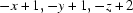; (vii) 
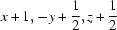
; (viii) 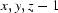.

**Fig. 1.**
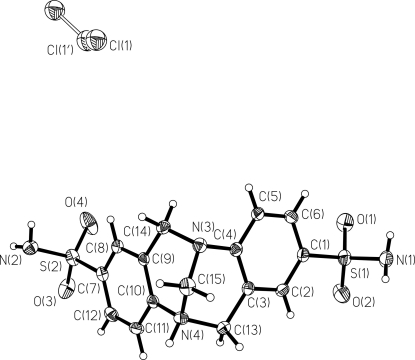
The structure of the title compound, showing displacement ellipsoids drawn at the 30% probability level.

**Fig. 2.**
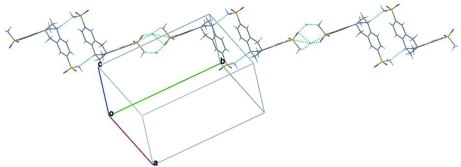
The one-dimensional doublechain formed through CH—O, and CH2—O interactions running along the a axis direction.

## supplementary crystallographic information

### Comment

Organic salts based on hydrogen bonding are a research field receiving great attention in recent years. As an extension of our study concentrating on hydrogen bonded assembly of organic acid and organic base (Jin *et al.*, 2010), herein we report the crystal structure of 2,9-Dibenzenesulfonamide-1,5-diazaium-bicyclo[3.3.1]dimethanodibenzo chloride.

The crystal of the title compound of the formula C_15_H_17_ClN_4_O_4_S_2_ was obtained by recrystallization of 2,9-Dibenzenesulfonamide-1,5-diazaium- bicyclo[3.3.1]dimethanodibenzo from methanol and HCl solution.

The compound is a salt. The asymmetric unit of the compound consists of one monocation, and one chloride anion (Fig. 1), respectively.

The chloride anion is disordered over two positions with occupancies of 0.78, and 0.22 respectively. In the compound there is a mirror plane running through the C15 atom. Two cations formed dimers *via* the N—H···N hydrogen bond between the NH_+_ cation and the amino group with N—N separation of 2.922 (6) A%. The cation dimers were connected together *via* the chloride anion through CH—Cl, Cl—O, and N—H···Cl contacts to form one-dimensional chain (A) running along the *b* axis direction (Fig. 2). Such adjacent chains were combined together by the CH_2_—O, and CH—O interactions to form two-dimensional sheet extending along the *bc* plane. Such kind of sheets were stacked along the *a* axis direction *via* the CH2—Cl interactions to form three-dimensional network structure. It is worthy to note that there are one-dimensional chains (B) that are running through the three-dimensional network. And such kind of chains were connected with the three-dimensional network through the N—H···S, and N—H···O interactions.

### Experimental

A solution of 2,9-dibenzenesulfonamide-1,5-diazabicyclo[3.3.1]dimethanodibenzo (38 mg, 0.1 mmol) was dissolved in 5 ml of methanol and 1 ml of conc. HCl under continuous stirring. The solution was stirred for about 1 h at room temperature, then the solution was filtered into a test tube. The solution was left standing at room temperature for several days, colorless block crystals were isolated after slow evaporation of the solution in air at ambient temperature. The crystals were collected and dried in air to give the title compound.

### Refinement

Hydrogen atoms attached to the C atoms were placed in calculated positions with d(C—H) = 0.93–0.97 Å. Positions of the hydrogen atoms at the NH groups were located from the Fourier difference syntheses and refined independently. All *U*_iso_ values were restrained on *U*_eq_ values of the parent atoms.

### Crystal data

**Table:**

C_15_H_17_N_4_O_4_S_2_^+^·Cl^−^	*Z* = 4
*M**_r_* = 416.90	*F*(000) = 864
Monoclinic, *P*2_1_/*c*	*D*_x_ = 1.681 Mg m^−^^3^
Hall symbol: -P 2ybc	Mo *K*α radiation, λ = 0.71073 Å
*a* = 11.5247 (11) Å	µ = 0.52 mm^−^^1^
*b* = 18.5693 (16) Å	*T* = 298 K
*c* = 8.1489 (7) Å	Block, colorless
β = 109.177 (1)°	0.40 × 0.33 × 0.27 mm
*V* = 1647.1 (3) Å^3^	

### Data collection

**Table:**

Bruker SMART CCD diffractometer	2895 independent reflections
Radiation source: fine-focus sealed tube	1944 reflections with *I* > 2σ(*I*)
graphite	*R*_int_ = 0.042
phi and ω scans	θ_max_ = 25.0°, θ_min_ = 2.9°
Absorption correction: multi-scan (*SADABS*; Bruker, 2002)	*h* = −11→13
*T*_min_ = 0.815, *T*_max_ = 0.869	*k* = −21→22
8082 measured reflections	*l* = −9→5

### Refinement

**Table:**

Refinement on *F*^2^	Primary atom site location: structure-invariant direct methods
Least-squares matrix: full	Secondary atom site location: difference Fourier map
*R*[*F*^2^ > 2σ(*F*^2^)] = 0.068	Hydrogen site location: inferred from neighbouring sites
*wR*(*F*^2^) = 0.219	H-atom parameters constrained
*S* = 1.08	*w* = 1/[σ^2^(*F*_o_^2^) + (0.0953*P*)^2^ + 5.3108*P*] where *P* = (*F*_o_^2^ + 2*F*_c_^2^)/3
2895 reflections	(Δ/σ)_max_ < 0.001
239 parameters	Δρ_max_ = 0.52 e Å^−^^3^
0 restraints	Δρ_min_ = −0.96 e Å^−^^3^

### Special details

**Table:**

Geometry. All e.s.d.'s (except the e.s.d. in the dihedral angle between two l.s. planes) are estimated using the full covariance matrix. The cell e.s.d.'s are taken into account individually in the estimation of e.s.d.'s in distances, angles and torsion angles; correlations between e.s.d.'s in cell parameters are only used when they are defined by crystal symmetry. An approximate (isotropic) treatment of cell e.s.d.'s is used for estimating e.s.d.'s involving l.s. planes.
Refinement. Refinement of *F*^2^ against ALL reflections. The weighted *R*-factor *wR* and goodness of fit *S* are based on *F*^2^, conventional *R*-factors *R* are based on *F*, with *F* set to zero for negative *F*^2^. The threshold expression of *F*^2^ > σ(*F*^2^) is used only for calculating *R*-factors(gt) *etc*. and is not relevant to the choice of reflections for refinement. *R*-factors based on *F*^2^ are statistically about twice as large as those based on *F*, and *R*- factors based on ALL data will be even larger.

### Fractional atomic coordinates and isotropic or equivalent isotropic displacement parameters (Å^2^)

**Table:**

	*x*	*y*	*z*	*U*_iso_*/*U*_eq_	Occ. (<1)
Cl1	0.1069 (3)	0.02872 (18)	0.3829 (5)	0.0948 (11)	0.776 (6)
Cl1'	0.0764 (12)	0.0213 (7)	0.4728 (19)	0.0948 (11)	0.224 (6)
N1	1.2145 (4)	0.1555 (3)	0.6436 (7)	0.0489 (13)	
H1A	1.2289	0.1965	0.5969	0.073*	
H1B	1.1813	0.1236	0.5598	0.073*	
N2	0.2583 (4)	0.4134 (3)	0.6686 (6)	0.0436 (12)	
H2A	0.2080	0.4192	0.7302	0.065*	
H2B	0.2262	0.3815	0.5843	0.065*	
N3	0.6862 (4)	0.3219 (2)	0.2614 (5)	0.0342 (10)	
N4	0.7476 (4)	0.4335 (2)	0.4164 (6)	0.0350 (10)	
H4	0.7507	0.4818	0.3997	0.042*	
O1	1.0815 (4)	0.1016 (2)	0.7888 (6)	0.0581 (12)	
O2	1.1807 (4)	0.2171 (3)	0.8899 (6)	0.0684 (14)	
O3	0.4230 (4)	0.4292 (3)	0.9437 (5)	0.0658 (14)	
O4	0.3793 (6)	0.3081 (3)	0.8158 (8)	0.0860 (18)	
S1	1.12094 (13)	0.17065 (8)	0.74765 (18)	0.0425 (4)	
S2	0.38898 (14)	0.38395 (8)	0.79416 (19)	0.0440 (4)	
C1	0.9909 (5)	0.2165 (3)	0.6090 (7)	0.0349 (12)	
C2	0.9842 (5)	0.2910 (3)	0.6153 (7)	0.0356 (12)	
H2	1.0470	0.3169	0.6947	0.043*	
C3	0.8829 (4)	0.3271 (3)	0.5022 (7)	0.0325 (11)	
C4	0.7906 (4)	0.2870 (3)	0.3842 (6)	0.0318 (11)	
C5	0.7975 (5)	0.2124 (3)	0.3834 (7)	0.0361 (12)	
H5	0.7337	0.1861	0.3071	0.043*	
C6	0.8967 (5)	0.1769 (3)	0.4933 (7)	0.0381 (12)	
H6	0.9011	0.1270	0.4907	0.046*	
C7	0.4950 (5)	0.3985 (3)	0.6817 (7)	0.0336 (12)	
C8	0.4905 (4)	0.3543 (3)	0.5427 (7)	0.0328 (11)	
H8	0.4314	0.3183	0.5080	0.039*	
C9	0.5745 (4)	0.3639 (3)	0.4549 (6)	0.0288 (11)	
C10	0.6603 (4)	0.4199 (3)	0.5082 (6)	0.0301 (11)	
C11	0.6638 (5)	0.4636 (3)	0.6472 (7)	0.0371 (12)	
H11	0.7223	0.5000	0.6815	0.045*	
C12	0.5815 (5)	0.4538 (3)	0.7349 (7)	0.0394 (13)	
H12	0.5833	0.4834	0.8278	0.047*	
C13	0.8742 (5)	0.4079 (3)	0.5131 (8)	0.0394 (13)	
H13A	0.8962	0.4223	0.6339	0.047*	
H13B	0.9320	0.4302	0.4648	0.047*	
C14	0.5740 (5)	0.3144 (3)	0.3071 (7)	0.0352 (12)	
H14A	0.5664	0.2649	0.3404	0.042*	
H14B	0.5034	0.3254	0.2062	0.042*	
C15	0.7088 (5)	0.3973 (3)	0.2424 (7)	0.0421 (13)	
H15A	0.6348	0.4199	0.1664	0.051*	
H15B	0.7728	0.4026	0.1901	0.051*	

### Atomic displacement parameters (Å^2^)

**Table:**

	*U*^11^	*U*^22^	*U*^33^	*U*^12^	*U*^13^	*U*^23^
Cl1	0.093 (2)	0.091 (2)	0.107 (3)	−0.0023 (16)	0.0411 (18)	−0.0070 (19)
Cl1'	0.093 (2)	0.091 (2)	0.107 (3)	−0.0023 (16)	0.0411 (18)	−0.0070 (19)
N1	0.041 (3)	0.047 (3)	0.063 (3)	0.006 (2)	0.023 (2)	0.008 (2)
N2	0.046 (3)	0.049 (3)	0.037 (3)	−0.004 (2)	0.015 (2)	−0.007 (2)
N3	0.035 (2)	0.038 (2)	0.031 (2)	0.0010 (19)	0.0125 (18)	−0.0018 (19)
N4	0.037 (2)	0.027 (2)	0.043 (2)	−0.0031 (18)	0.016 (2)	0.0048 (19)
O1	0.061 (3)	0.058 (3)	0.058 (3)	0.007 (2)	0.023 (2)	0.023 (2)
O2	0.063 (3)	0.072 (3)	0.056 (3)	0.017 (2)	−0.001 (2)	−0.014 (2)
O3	0.051 (3)	0.113 (4)	0.034 (2)	0.005 (3)	0.015 (2)	−0.011 (2)
O4	0.115 (4)	0.051 (3)	0.127 (5)	0.026 (3)	0.087 (4)	0.040 (3)
S1	0.0381 (8)	0.0485 (9)	0.0396 (8)	0.0072 (6)	0.0111 (6)	0.0007 (6)
S2	0.0533 (9)	0.0450 (9)	0.0411 (8)	0.0103 (7)	0.0256 (7)	0.0109 (6)
C1	0.030 (3)	0.038 (3)	0.039 (3)	0.002 (2)	0.015 (2)	−0.001 (2)
C2	0.030 (3)	0.038 (3)	0.041 (3)	−0.006 (2)	0.013 (2)	−0.007 (2)
C3	0.030 (3)	0.035 (3)	0.037 (3)	−0.001 (2)	0.019 (2)	−0.003 (2)
C4	0.030 (3)	0.037 (3)	0.032 (3)	0.000 (2)	0.014 (2)	−0.003 (2)
C5	0.028 (3)	0.040 (3)	0.039 (3)	−0.001 (2)	0.010 (2)	−0.010 (2)
C6	0.041 (3)	0.031 (3)	0.047 (3)	−0.002 (2)	0.020 (3)	−0.005 (2)
C7	0.036 (3)	0.034 (3)	0.033 (3)	0.009 (2)	0.015 (2)	0.004 (2)
C8	0.027 (3)	0.033 (3)	0.035 (3)	0.003 (2)	0.007 (2)	0.001 (2)
C9	0.026 (2)	0.028 (3)	0.030 (3)	0.006 (2)	0.005 (2)	0.002 (2)
C10	0.030 (3)	0.026 (3)	0.031 (3)	0.006 (2)	0.005 (2)	0.004 (2)
C11	0.041 (3)	0.027 (3)	0.041 (3)	−0.004 (2)	0.011 (2)	−0.005 (2)
C12	0.049 (3)	0.035 (3)	0.033 (3)	0.007 (2)	0.012 (2)	−0.004 (2)
C13	0.031 (3)	0.035 (3)	0.052 (3)	−0.003 (2)	0.013 (2)	0.000 (2)
C14	0.030 (3)	0.039 (3)	0.034 (3)	−0.002 (2)	0.007 (2)	−0.007 (2)
C15	0.047 (3)	0.048 (3)	0.036 (3)	0.003 (3)	0.020 (3)	0.007 (3)

### Geometric parameters (Å, °)

**Table:**

Cl1'—Cl1'^i^	2.11 (3)	C2—H2	0.9300
N1—S1	1.600 (5)	C3—C4	1.392 (7)
N1—H1A	0.8900	C3—C13	1.509 (7)
N1—H1B	0.8900	C4—C5	1.388 (7)
N2—S2	1.613 (5)	C5—C6	1.367 (7)
N2—H2A	0.8900	C5—H5	0.9300
N2—H2B	0.8900	C6—H6	0.9300
N3—C15	1.441 (7)	C7—C8	1.386 (7)
N3—C4	1.442 (6)	C7—C12	1.397 (8)
N3—C14	1.465 (6)	C8—C9	1.391 (7)
N4—C10	1.460 (6)	C8—H8	0.9300
N4—C13	1.490 (7)	C9—C10	1.401 (7)
N4—C15	1.499 (7)	C9—C14	1.513 (7)
N4—H4	0.9100	C10—C11	1.384 (7)
O1—S1	1.437 (5)	C11—C12	1.375 (7)
O2—S1	1.427 (5)	C11—H11	0.9300
O3—S2	1.425 (5)	C12—H12	0.9300
O4—S2	1.428 (5)	C13—H13A	0.9700
S1—C1	1.770 (5)	C13—H13B	0.9700
S2—C7	1.772 (5)	C14—H14A	0.9700
C1—C2	1.387 (7)	C14—H14B	0.9700
C1—C6	1.392 (7)	C15—H15A	0.9700
C2—C3	1.398 (7)	C15—H15B	0.9700
			
S1—N1—H1A	109.3	C4—C5—H5	119.5
S1—N1—H1B	109.2	C5—C6—C1	119.2 (5)
H1A—N1—H1B	109.5	C5—C6—H6	120.4
S2—N2—H2A	109.2	C1—C6—H6	120.4
S2—N2—H2B	109.2	C8—C7—C12	121.3 (5)
H2A—N2—H2B	109.5	C8—C7—S2	119.1 (4)
C15—N3—C4	111.8 (4)	C12—C7—S2	119.6 (4)
C15—N3—C14	109.0 (4)	C7—C8—C9	120.0 (5)
C4—N3—C14	112.7 (4)	C7—C8—H8	120.0
C10—N4—C13	113.2 (4)	C9—C8—H8	120.0
C10—N4—C15	111.4 (4)	C8—C9—C10	118.3 (4)
C13—N4—C15	107.1 (4)	C8—C9—C14	120.7 (4)
C10—N4—H4	108.3	C10—C9—C14	121.0 (4)
C13—N4—H4	108.3	C11—C10—C9	121.2 (5)
C15—N4—H4	108.3	C11—C10—N4	118.5 (4)
O2—S1—O1	117.1 (3)	C9—C10—N4	120.3 (4)
O2—S1—N1	107.9 (3)	C12—C11—C10	120.5 (5)
O1—S1—N1	106.7 (3)	C12—C11—H11	119.8
O2—S1—C1	108.0 (3)	C10—C11—H11	119.8
O1—S1—C1	108.2 (3)	C11—C12—C7	118.7 (5)
N1—S1—C1	108.8 (3)	C11—C12—H12	120.6
O3—S2—O4	119.1 (3)	C7—C12—H12	120.6
O3—S2—N2	106.3 (3)	N4—C13—C3	111.0 (4)
O4—S2—N2	108.5 (3)	N4—C13—H13A	109.4
O3—S2—C7	108.0 (3)	C3—C13—H13A	109.4
O4—S2—C7	108.0 (3)	N4—C13—H13B	109.4
N2—S2—C7	106.2 (2)	C3—C13—H13B	109.4
C2—C1—C6	120.7 (5)	H13A—C13—H13B	108.0
C2—C1—S1	120.1 (4)	N3—C14—C9	111.8 (4)
C6—C1—S1	119.1 (4)	N3—C14—H14A	109.3
C1—C2—C3	119.9 (5)	C9—C14—H14A	109.3
C1—C2—H2	120.1	N3—C14—H14B	109.3
C3—C2—H2	120.1	C9—C14—H14B	109.3
C4—C3—C2	118.9 (5)	H14A—C14—H14B	107.9
C4—C3—C13	121.5 (5)	N3—C15—N4	110.0 (4)
C2—C3—C13	119.6 (5)	N3—C15—H15A	109.7
C5—C4—C3	120.3 (5)	N4—C15—H15A	109.7
C5—C4—N3	118.8 (4)	N3—C15—H15B	109.7
C3—C4—N3	120.9 (5)	N4—C15—H15B	109.7
C6—C5—C4	121.0 (5)	H15A—C15—H15B	108.2
C6—C5—H5	119.5		
			
O2—S1—C1—C2	20.7 (5)	C12—C7—C8—C9	1.1 (7)
O1—S1—C1—C2	148.3 (4)	S2—C7—C8—C9	−178.7 (4)
N1—S1—C1—C2	−96.2 (5)	C7—C8—C9—C10	−1.5 (7)
O2—S1—C1—C6	−159.9 (4)	C7—C8—C9—C14	177.7 (5)
O1—S1—C1—C6	−32.2 (5)	C8—C9—C10—C11	1.5 (7)
N1—S1—C1—C6	83.3 (5)	C14—C9—C10—C11	−177.7 (5)
C6—C1—C2—C3	−1.2 (8)	C8—C9—C10—N4	−178.0 (4)
S1—C1—C2—C3	178.2 (4)	C14—C9—C10—N4	2.8 (7)
C1—C2—C3—C4	−0.3 (7)	C13—N4—C10—C11	76.1 (6)
C1—C2—C3—C13	178.2 (5)	C15—N4—C10—C11	−163.2 (5)
C2—C3—C4—C5	2.1 (7)	C13—N4—C10—C9	−104.4 (5)
C13—C3—C4—C5	−176.4 (5)	C15—N4—C10—C9	16.4 (6)
C2—C3—C4—N3	−178.1 (4)	C9—C10—C11—C12	−1.0 (8)
C13—C3—C4—N3	3.4 (7)	N4—C10—C11—C12	178.5 (5)
C15—N3—C4—C5	−162.8 (4)	C10—C11—C12—C7	0.5 (8)
C14—N3—C4—C5	74.0 (6)	C8—C7—C12—C11	−0.5 (8)
C15—N3—C4—C3	17.5 (6)	S2—C7—C12—C11	179.3 (4)
C14—N3—C4—C3	−105.8 (5)	C10—N4—C13—C3	75.6 (5)
C3—C4—C5—C6	−2.5 (8)	C15—N4—C13—C3	−47.6 (5)
N3—C4—C5—C6	177.7 (5)	C4—C3—C13—N4	13.2 (7)
C4—C5—C6—C1	1.0 (8)	C2—C3—C13—N4	−165.3 (4)
C2—C1—C6—C5	0.9 (8)	C15—N3—C14—C9	−48.8 (5)
S1—C1—C6—C5	−178.6 (4)	C4—N3—C14—C9	76.0 (5)
O3—S2—C7—C8	172.3 (4)	C8—C9—C14—N3	−166.0 (4)
O4—S2—C7—C8	42.2 (5)	C10—C9—C14—N3	13.3 (6)
N2—S2—C7—C8	−74.1 (5)	C4—N3—C15—N4	−54.7 (5)
O3—S2—C7—C12	−7.5 (5)	C14—N3—C15—N4	70.6 (5)
O4—S2—C7—C12	−137.6 (5)	C10—N4—C15—N3	−52.9 (6)
N2—S2—C7—C12	106.1 (4)	C13—N4—C15—N3	71.4 (5)

Symmetry codes: (i) −*x*, −*y*, −*z*+1.

### Hydrogen-bond geometry (Å, °)

**Table:**

*D*—H···*A*	*D*—H	H···*A*	*D*···*A*	*D*—H···*A*
N1—H1A···O2^ii^	0.89	2.26	3.081 (8)	153
N1—H1B···Cl1^iii^	0.89	2.26	3.141 (7)	170
N2—H2A···Cl1^iv^	0.89	2.19	3.042 (6)	160
N2—H2B···O1^v^	0.89	2.46	3.109 (7)	130
N2—H2B···O2^v^	0.89	2.36	3.240 (7)	168
N4—H4···N2^vi^	0.91	2.02	2.922 (7)	173
C12—H12···O3^vii^	0.93	2.49	3.416 (7)	175
C13—H13A···Cl1^viii^	0.97	2.76	3.514 (7)	135
C14—H14A···O4^ii^	0.97	2.50	3.212 (9)	130
C15—H15A···O3^ix^	0.97	2.52	3.443 (7)	158

Symmetry codes: (ii) *x*, −*y*+1/2, *z*−1/2; (iii) *x*+1, *y*, *z*; (iv) *x*, −*y*+1/2, *z*+1/2; (v) *x*−1, −*y*+1/2, *z*−1/2; (vi) −*x*+1, −*y*+1, −*z*+1; (vii) −*x*+1, −*y*+1, −*z*+2; (viii) *x*+1, −*y*+1/2, *z*+1/2; (ix) *x*, *y*, *z*−1.
